# Retrospective analysis of drug resistance characteristics and infection related risk factors of multidrug-resistant organisms (MDROs) isolated from the orthopedics department of a tertiary hospital

**DOI:** 10.1038/s41598-023-28270-3

**Published:** 2023-02-07

**Authors:** Xiaowei Yang, Runsheng Guo, Bi Zhang, Banglin Xie, Song Zhou, Bin Zhang, Qi Lai

**Affiliations:** 1 Department of Orthopedics, First Affiliated Hospital of Nanchang University, No. 17 Yong Wai Zheng Street, Nanchang, 330006 Jiangxi China; 2 Department of Spine Surgery, Ganzhuo People’s Hospital, No.16 MeiGuan Road, Zhanggong District, Ganzhou City, 341000 Jiangxi China

**Keywords:** Diseases, Risk factors

## Abstract

Patients infected with multidrug-resistant organisms (MDROs) are known to exhibit longer hospital stays and a significantly poorer prognosis. We performed a 6-year retrospective analysis of nosocomial infections reported in the orthopedics department of our institution, to gain valuable insights into antibiotic sensitivity and infectious characteristics of MDROs, in order to deduce effective measures to control the occurrence of multidrug-resistant infections in clinical practice. A retrospective, single center surveillance study (January 2012–December 2017) was performed on the nosocomial infections recorded in the department of orthopedics. A nosocomial infection is defined as one that develops when a patient is residing in a hospital but was not present at the time of admission. All relevant data, including basic patient information, cultivated bacterial strains, and antimicrobial resistance, was obtained from the hospital information system. A total of 1392 strains of pathogenic bacteria were isolated; 358 belonged to MDROs (detection rate = 25.7%). All the isolated strains of MDROs were mostly from secretions (52.2%). The number of cases infected with MDROs were 144 (40.2%) and 129 (36.0%) in the trauma and spinal wards, respectively. MRSA showed high resistance to β lactam antibiotics, but was sensitive to quinolone antibiotics, linezolid and cotrimoxazole. ESBL-positive strains showed more sensitivity to carbapenem antibiotics (resistance rate < 10%). MDR nonfermenters showed high resistance to most antibiotics. Logistic multivariate analysis revealed age, open injury, and central nervous system injury as independent risk factors for multidrug resistant infections. A high antibiotic resistance rate and an increasing prevalence of infections with MDROs was identified in the orthopedics department. Patients with open injury, central nervous system injury and those aged ≥ 60 years, were more prone to multidrug-resistant infections. Clinicians should pay more attention to such patients in order to actively prevent and control the occurrence of infections caused by MDROs.

## Introduction

Hospital-acquired infections (HAIs), especially at surgical sites, are catastrophic complications that lead to higher in-hospital mortality, longer hospitalization, and greater healthcare expenditure^1,2^. With the rapid development of invasive medical devices, the contribution of HAI to surgery-related morbidity and mortality has increased considerably^3^. This effect is particularly noticeable in orthopedics, a medical specialty that is highly reliant on implants (e.g., internal fixation devices for bone fractures), wound dressings, and catheters.

Antibiotics act as the nemesis of bacterial infections and since the discovery of penicillin in 1928, infection related disability and death rate have greatly reduced, thereby significantly improving patients’ quality of life^4^. However, due to drug abuse stemming from hospital over-prescription as well as excessive self-medication, antibiotic resistance among pathogens has become a serious problem^5–7^. The continuous exposure to different antibiotics, owing to their indiscriminate clinical application has led to the cumulative acquisition of resistant traits in major pathogens, giving rise to the multidrug-resistant organisms (MDROs). These are defined as pathogenic strains that are resistant to three or more antibiotic classes at the same time and include methicillin-resistant *Staphylococcus aureus* (MRSA), Extended-spectrum β-lactamase (ESBL)-positive *Escherichia coli*, ESBL-positive *Klebsiella pneumoniae*, multidrug-resistant *Acinetobacter baumannii* (MDR-AB), multidrug-resistant *Pseudomonas aeruginosa* (MDR-PA), *Enterococcus* and *Enterobacter*. Compared with antibiotic susceptible strains, patients infected with MDROs display a significantly longer hospital stay and a markedly poorer prognosis^8,9^. It has been revealed that MDROs have now become the principal causative pathogens for nosocomial infections^10^. In the United States, nearly two million patients are reported to develop hospital-acquired infection per year and most of these infections are the result of MDROs^11^. MDROs can cause different types of infections, including pneumonia, urinary tract infection, abdominal infection and surgical site infection, leading to longer hospital stays, increased mortality, and higher hospitalization costs^1,12,13^. Therefore, understanding the resistance patterns and clinical distribution of MDROs is imperative in order to develop effective prevention and control measures aimed at avoiding the outbreak of MDROs in clinical practice.

In the present study, we collected data for all cases of nosocomial infections reported in the orthopedics department, from 2012 to 2017. Among these, we screened out the cases of infections caused by MDROs, and analyzed their clinical characteristics, distribution and drug resistance patterns, as well as the main risk factors for multidrug-resistant infections. Through this research, we aim to gain a better understanding of MDROs and to devise effective measures to control the occurrence of multidrug-resistant infections in future.

## Methods

### Location and study design

This study was conducted in the department of orthopedics of the first affiliated hospital of Nanchang University. Over 10,000 surgeries are performed annually in this hospital, of which 60% are level III and IV. The sickbed utilization rate is greater than 130%.

A retrospective, single center surveillance study (January 2012–December 2017) was performed on the nosocomial infections recorded in the department of orthopedics. A nosocomial infection is defined as one acquire infection during the process of receiving health care that was not present during the time of admission^14^. In this study, we specifically classified infections as nosocomial if they occurred 48 h post-admission/post-surgery or later. Furthermore, bacteria that exhibited resistance to three or more class of antibiotic was defined as MDROs, and those nosocomial infections that were caused by such bacteria were screened out of the initially identified cases. Two researchers collected all relevant data, including basic patient information, cultivated bacterial strains, and antimicrobial resistance, from the Hospital Information Warehouse and Clinical Microbiology Laboratory.

### Strain identification and antibiotic-sensitivity testing

All clinical specimens, including secretions, urine, blood, joint fluids, and cerebrospinal fluid obtained from the orthopedics department between January 2012 and December 2017 were included in the analysis if they tested positive for pathogens. Identical strains from the same patient were excluded. The collected specimens were stored in sterile culture tubes and sent to the microbiology laboratory within 2 h of collection. The different bacterial strains and corresponding antibacterial sensitivities were identified using the VITEK-2 automated system (bioMérieux Inc., France). According to the updated guidelines from the Clinical and Laboratory Standards Institute (CLSI), antimicrobial susceptibility was tested with the Kirby–Bauer method and minimum inhibitory concentrations. *Staphylococcus aureus* ATCC29213, *E. coli* ATCC25922, *K. pneumoniae* ATCC35657 and *P. aeruginosa* ATCC27853 strains were used in the antimicrobial susceptibility tests for quality control. Phenotypic confirmatory tests for ESBL-positive *E. coli* and MRSA were performed according to the latest CLSI guidelines^15^. All methods were performed in accordance with the relevant guidelines and regulations.

### Statistical analysis

Pertinent data were collected using the Word Processing System. Trend analysis and between-group differences were determined using the Chi-square test. Significant risk factors were screened out according to the results of the Chi-square test, and further risk factor analysis was performed via binomial logistic regression analysis. All statistical analyses were performed using SPSS software version 23.0 (SPSS Inc., Chicago, IL, USA) and statistical significance was set at *P* < 0.05.

### Ethics approval and consent to participate

The study protocol was approved by the Ethical Institutional Review Board of the First Affiliated Hospital of Nanchang University, and written informed consent was obtained from all study participants.

## Results

### Detection rate of MDROs

**Figure 1:**
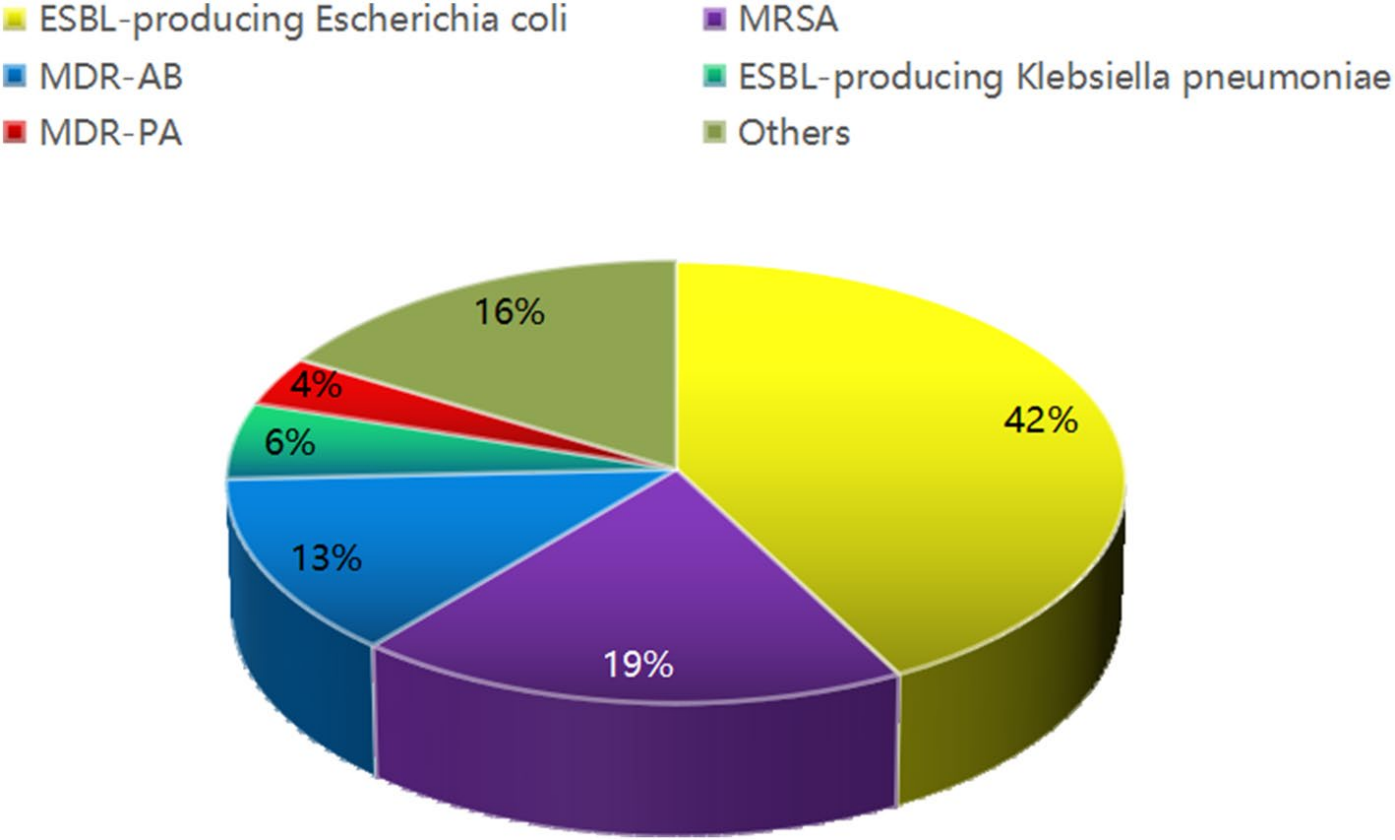
Composition of MDROs.

**Figure 2:**
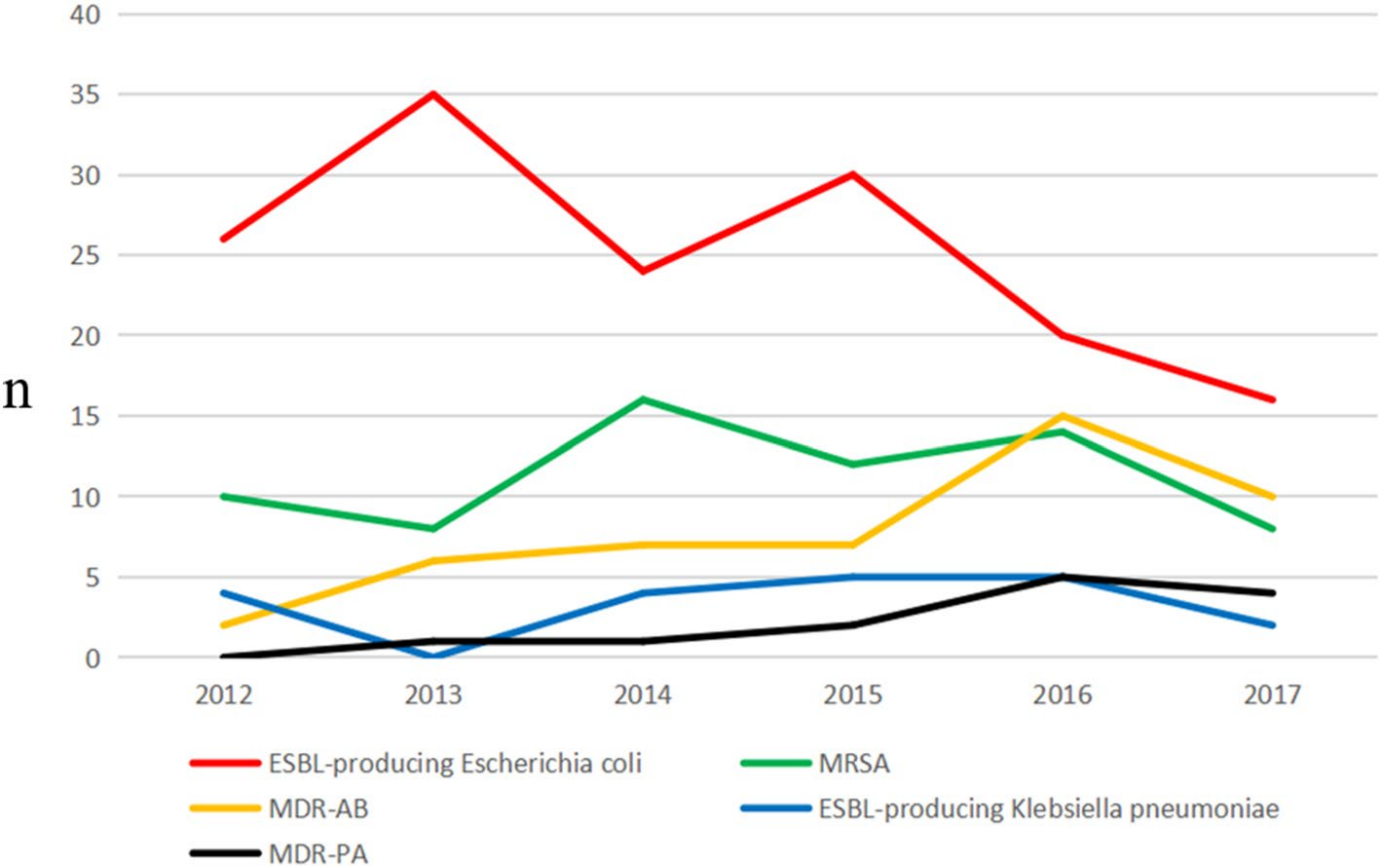
The trend of detection rates of different MDROs from 2012 to 2017.

From 2012 to 2017, a total of 1392 strains of pathogenic bacteria were isolated, among which 358 strains belonged to MDROs, resulting in a detection rate of 25.7%. As shown in Fig. 1, the main pathogenic bacteria identified in this study were as follows: 151 strains of ESBL-positive *E. coli* (42.2%), 68 strains of MRSA (19.0%), 47 strains of MDR-AB (13.1%), 20 strains of ESBL-positive *K. pneumoniae* (5.6%), and 13 strains of MDR-PA (3.6%). Figure 2 depicts the trend of detection rates of different MDROs from 2012 to 2017. During the study period of 6 years, the prevalence of ESBL-positive *E. coli* decreased, while that of MDR nonfermenters, including MDR-AB and MDR-PA, increased.

### Distribution of bacterial strains

#### Distribution on the basis of specimen

As shown in Table 1, the 358 isolated strains of MDROs were mostly from secretions (52.2%), followed by urine (29.1%), sputum (10.3%), blood (6.1%), joint fluid (1.1%) and cerebrospinal fluid (0.6%). The MDROs isolated from urine were mainly ESBL-positive *E. coli*, while the most common strain isolated from secretions was MRSA, followed by ESBL-positive *E. coli.* MDR-AB were the predominant pathogens in the sputum specimens.

**Table 1:** Distribution of MDROs (n, %).

Specimen	ESBL-producing *Escherichia coli*	MRSA	MDR-AB	ESBL-producing *Klebsiella pneumoniae*	MDR-PA	Others	Total
Secretions	50	59	26	11	7	34	187 (52.2)
Urine	81	2	3	3	0	15	104 (29.1)
Sputum	4	5	14	5	5	4	37 (10.3)
Blood	13	1	1	1	1	5	22 (6.1)
Joint fluid	1	1	1	0	0	1	4 (1.1)
Cerebrospinal fluid	1	0	1	0	0	0	2 (0.6)
Others	1	0	1	0	0	0	2 (0.6)
Total	151	68	47	20	13	59	358

#### Distribution according to the different areas of orthopedics

**Figure 3:**
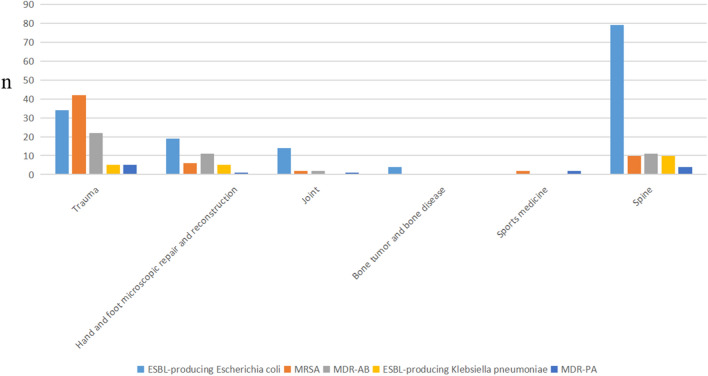
The distribution of the various MDROs in each ward.

**Figure 4:**
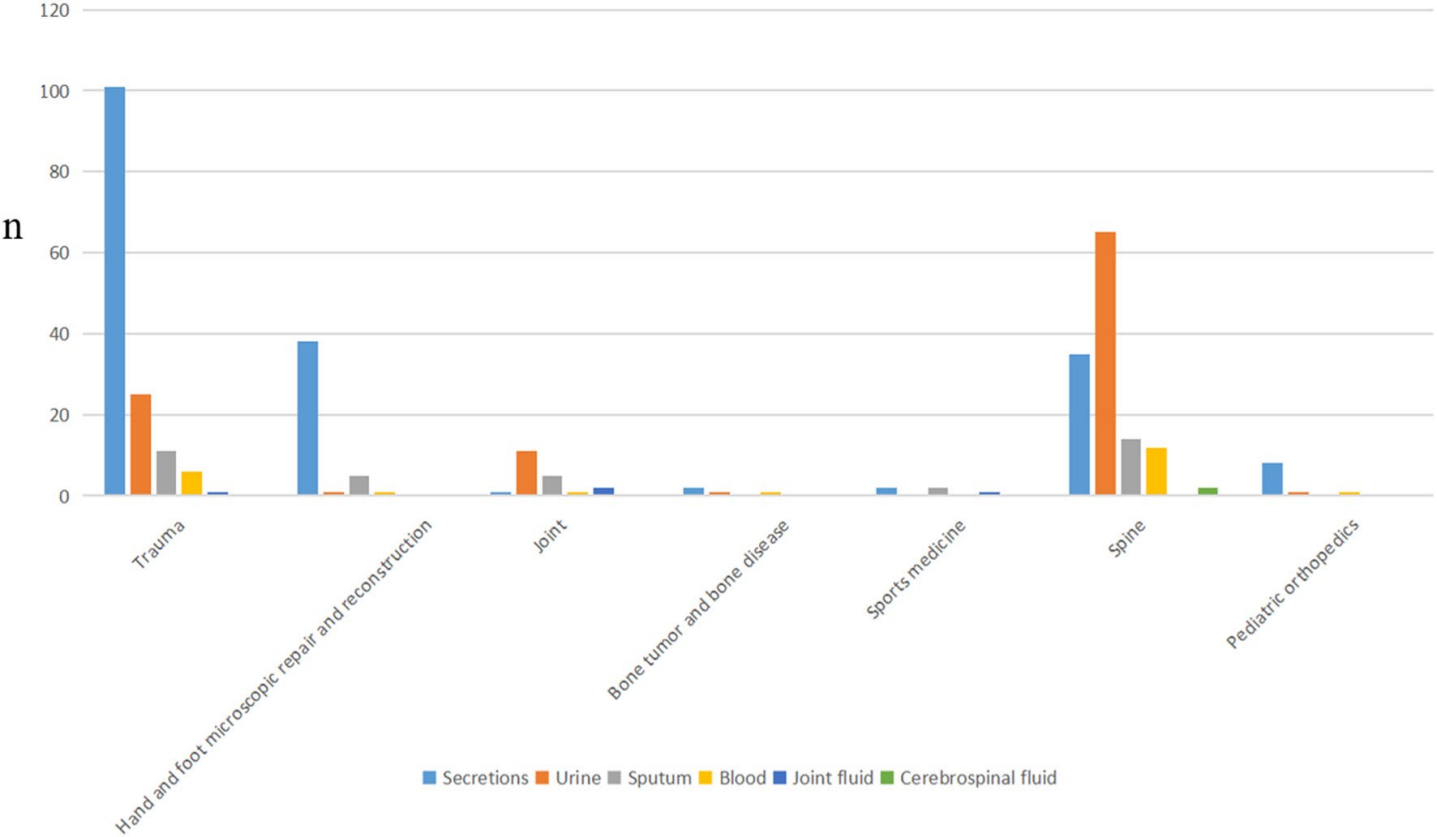
The source and distribution of specimens in each orthopedic ward.

In total, MDROs were isolated from 144 cases (40.2%) in the trauma ward, followed by 129 cases (36.0%) in spinal surgery, 46 cases (12.8%) in hand and foot microsurgery, 20 cases (5.6%) in joint surgery (5.6%), 10 cases (2.8%) in pediatric orthopedic ward, 5 cases (1.4%) in sports medicine, and 4 cases (1.1%) in bone oncology. The distribution of the various MDROs in each ward is shown in Fig. 3. It can be seen that MRSA was mainly distributed in the trauma ward, spinal surgery ward and pediatric orthopedic ward, while MDR-AB was mainly distributed in the trauma ward, hand-foot microsurgery and spinal surgery ward. ESBL-positive *E. coli* was widely found in all departments, with the highest proportion in the spinal surgery ward. The source and distribution of specimens in each orthopedic ward are shown in Fig. 4. The primary infection sites in patients from both the trauma ward (70.1%) and the hand and foot microsurgery ward (82.6%) were skin and soft tissues (mainly derived from secretions), whereas urinary tract infections were predominant in the spinal surgery (50.4%) and joint surgery (55%) departments.

### Drug resistance analysis of the chiefly isolated MDROs

#### MRSA

From 2012 to 2017, 68 strains of MRSA were isolated, accounting for 19.0% of all MDROs. As shown in Table 2, all MRSA strains were resistant to penicillin, ceftriaxone and ampicillin, and more than 80% of strains were resistant to amoxicillin/clavulanic acid, ampicillin/sulbactam and oxacillin. By contrast, these strains did demonstrate a low resistance (< 30%) to quinolone antibiotics like levofloxacin, moxifloxacin and ciprofloxacin. Furthermore, all MRSA strains showed high sensitivity to linezolid and cotrimoxazole, with drug resistance rates lower than 10%. Few MRSA strains were also found to be resistant to tigecycline, nitrofurantoin and vancomycin.

**Table 2:** Drug-resistance rate of MRSA in orthopaedics from 2012 to 2017.

Antibiotics	Total strains	Resistant strains	Drug resistance rate [% (strains/strains)]
Penicillin	51	51	100.0
Amoxicillin-clavulanic acid	50	46	92.0
Ceftriaxone	45	45	100.0
Levofloxacin	62	16	25.8
Tetracycline	64	30	46.9
Moxifloxacin	54	7	13.0
Ciprofloxacin	61	17	27.9
Gentamicin	67	25	37.3
Cotrimoxazole	67	6	9.0
Tigecycline	10	0	0
Ampicillin/sulbactam	42	40	95.2
Ampicillin	49	49	100.0
Clindamycin	63	50	79.4
Erythromycin	63	50	79.4
Linezoelid	66	2	3.0
Rifampicin	67	12	17.9
Quinupristin/Dalfopristin	67	7	10.4
Nitrofurantoin	17	0	0
Vancomycin	60	0	0

#### ESBL-positive strains

A total of 151 strains (42.2%) of ESBL-positive *E. coli* were isolated from 2012 to 2017, which accounted for the highest proportion of all MDROs. Meanwhile, twenty strains (5.6%) of ESBL-positive *K. pneumoniae* were isolated. As shown in Table 3, the drug resistance of ESBL-positive *K. pneumoniae* was generally higher than that of ESBL-positive *E. coli*. ESBL-positive *E. coli* showed high resistance to cefazolin, ceftriaxone and ampicillin (more than 90%), but low resistance to piperacillin/tazobactam (6.5%) and cefoperazone/sulbactam (8.4%). The resistance to quinolone antibiotics such as levofloxacin and ciprofloxacin was about 50%. These strains were more sensitive to carbapenem antibiotics such as ertapenem and imipenem with a drug resistance rate of less than 10%. Furthermore, we found that all strains of ESBL-positive *K. pneumoniae* were resistant to ceftriaxone and ampicillin, while they showed low resistance to ertapenem, imipenem and meropenem, ranging from 28.6 to 38.9%.

**Table 3:** Drug-resistance rate of ESBL-producing strains from 2012 to 2017.

Antibiotics	ESBL-positive *Escherichia coli*			ESBL-positive *Klebsiella pneumoniae*		
	Total strains	Resistant strains	Drug resistance rate [% (strains/strains)]	Total strains	Resistant strains	Drug resistance rate [% (strains/strains)]
Amikacin	135	11	8.1	20	8	40.0
Cefazolin	114	114	100.0	13	13	100.0
Ceftriaxone	115	115	100.0	13	13	100.0
Levofloxacin	137	60	43.8	20	10	50.0
Tetracycline	57	36	63.2	13	12	92.3
Tobramycin	123	38	30.9	20	12	60.0
Cefoperazone/sulbactam	107	9	8.4	16	6	37.5
Ciprofloxacin	136	72	52.9	20	10	50.0
Gentamicin	136	68	50.0	20	15	75.0
Meropenem	123	13	10.6	18	7	38.9
Cotrimoxazole	140	85	60.7	20	13	65.0
Ertapenem	112	10	8.9	14	4	28.6
Imipenem	137	9	6.6	20	7	35.0
Ampicillin/sulbactam	80	54	67.5	7	7	100.0
Ampicillin	137	137	100.0	20	20	100.0
Aztreonam	116	66	56.9	13	11	84.6
Peracillin/tazobar	138	9	6.5	20	8	40.0
Nitrofurantoin	78	2	2.6	6	2	33.3

#### MDR nonfermenters

Nonfermentative strains isolated from the specimens were mainly MDR-AB and MDR-PA, accounting for 13.1% and 3.6% of all MDROs, respectively. As shown in Table 4, these two strains exhibited high resistance to most antibiotics, and the overall drug resistance of MDR-AB was higher than that of MDR-PA. Both strains showed the highest resistance to β-lactam antibiotics like cefazolin, ceftriaxone, ceftazidime and ampicillin, reaching a drug resistance rate of 100%. The resistance rates of MDR-AB to levofloxacin and ciprofloxacin were 78.7% and 100%, respectively. By contrast, MDR-PA showed lower resistant rates to these two antibiotics, which were merely 46.2% and 53.8%, respectively. Although, MDR-AB and MDR-PA had the lowest resistance rates to cefoperazone/sulbactam (31.7%) and amikacin (41.7%), respectively, these were still within the high range of drug resistance.

**Table 4:** Drug-resistance rate of nonfermentative MDROs in orthopaedics from 2012 to 2017.

Antibiotics	MDR-AB			MDR-PA		
	Total strains	Resistant strains	Drug resistance rate [% (strains/strains)]	Total strains	Resistant strains	Drug resistance rate[% (strains/strains)]
Cefotetan	43	43	100.0	9	9	100.0
Amikacin	29	21	72.4	12	5	41.7
Cefazolin	40	40	100.0	9	9	100.0
Ceftriaxone	44	44	100.0	12	12	100.0
Cefepime	45	44	97.8	12	9	75.0
Levofloxacin	47	37	78.7	13	6	46.2
Tetracycline	31	26	83.9	3	2	66.7
Tobramycin	45	36	80.0	11	6	54.5
Cefoperazone/sulbactam	41	13	31.7	7	3	42.9
Ceftazidime	45	45	100.0	12	10	83.3
Ciprofloxacin	45	45	100.0	13	7	53.8
Gentamicin	47	45	95.7	13	6	46.2
Meropenem	44	42	95.5	6	4	66.7
Cotrimoxazole	47	37	78.7	11	11	100.0
Imipenem	42	34	81.0	12	9	75.0
Ampicillin/sulbactam	43	35	81.4	6	6	100.0
Ampicillin	40	40	100.0	7	7	100.0
Aztreonam	36	34	94.4	7	7	100.0
Peracillin/tazobar	42	35	83.3	12	10	83.3
Nitrofurantoin	39	37	94.9	8	8	100.0

### Risk factor analysis of multidrug-resistant infections

As shown in Table 5, the following factors were investigated: basic clinical characteristics (age, gender), personal history (smoking, drinking), underlying diseases (diabetes, hypertension), injury type (open injury, central nervous injury), blood biochemistry (albumin level, hemoglobin level). The results showed that patients with old age, open injury and central nerve injury were more liable to be infected with MDROs (*P* < 0.05). As shown in Table 6, the results of logistic multivariate analysis showed that age [OR = 1.838, 95% CI (1.390–2.429), *P* < 0.05], open injury [OR = 1.737, 95% CI (1.335–2.259), *P* < 0.05], central nerve injury [OR = 2.821, 95% CI (2.020–3.940), *P* < 0.05] were the independent risk factors for multidrug-resistant infections.

**Table 5:** Univariate analysis of risk factors for MDROs.

	Non-MDROs (n = 1034)	MDROs (n = 358)	χ^2^	*P*
Age (years)				
< 60	809	244	14.677	0.000
≥ 60	225	114		
Gender				
Male	732	262	0.745	0.388
Female	302	96		
Smoking history (n, %)	71 (6.9)	31 (8.7)	1.259	0.262
Alcohol history (n, %)	32 (3.1)	15 (4.2)	0.978	0.323
Diabetes (n, %)	65 (6.3)	29 (12.3)	1.390	0.238
Hypertension (n, %)	86 (8.3)	42 (11.7)	3.713	0.054
Central lesion (n, %)	105 (10.2)	79 (22.1)	32.896	0.000
Open injury (n, %)	361 (34.9)	150 (41.9)	5.587	0.018
Duration of hospital stays > 15 days (n, %)	894 (79.2)	316 (92.2)	0.765	0.382
Albumin < 30 g/L (n, %)	268 (25.9)	79 (22.1)	2.108	0.147
Hemoglobin < 90 g/L (n, 10%)	201 (19.4)	78 (21.8)	0.915	0.339

**Table 6:** Logistic multivariate analysis of MDROS infection.

Risk factor	β value	SE value	Ward χ^2^	*P* value	OR value	95%CI
Age	0.608	0.142	18.262	< 0.05	1.838	1.390–2.429
Open injuries	0.552	0.134	16.919	< 0.05	1.737	1.335–2.259
Central lesion	1.037	0.170	37.037	< 0.05	2.821	2.020–3.940

## Discussion

Infection is a commonly encountered complication in orthopedic patients^16,17^. With the continuous development of science and technology, the frequency of traffic and construction site accidents as well as the proportion of open and multiple injuries has increased significantly, leading to an escalation of nosocomial infections encountered in the inpatient departments of hospitals^18,19^. In addition, in recent years, due to the overuse of antibiotics, MDROs have now become the primary cause of nosocomial infections and this not only increases the economic burden on the patient, but also the difficulty of treatment^20^. In our study, a total of 1392 strains of pathogenic bacteria were isolated from the department of orthopedics from 2012 to 2017, including 358 strains of MDROs, with a constituent ratio of 25.7%, which is similar to some orthopedic units in Europe^21^. As for the composition of MDROs in our study, these were largely composed of gram-negative bacteria, including ESBL-positive *E. coli* (151 strains, 42.2%), MRSA (68 strains, 19.0%), MDR-AB (47 strains, 13.1%), ESBL-producing *K. pneumoniae* (20 strains, 5.6%), MDR-PA (13 strains, 3.6%), consistent with the data reported in a previous study conducted in north China^22^. However, another similar research conducted in the Australian hospitals indicated MRSA as the predominant MDRO^23^. This apparent discrepancy might be attributed to the difference in the procedures and tissue samples between the two studies. In their study, Vickers et al.^23^ only surveyed pathogens isolated from incisional secretions, but in our study, we surveyed pathogens isolated from a much larger variety of samples, including urine, sputum and blood, which were more likely to infected by gram-negative bacteria^24–27^. The findings of our study indicate that it is critical to implement effective measures to control infections caused by gram-negative MDROs in hospitalized patients. Besides, among the isolated gram-negative MDROs, the prevalence of MDR nonfermenters (MDR-AB and MDR-PA) in our study showed a rising trend from 2012 to 2017, a finding that needs to be paid special attention.

The infection rate of MDROs in trauma patients has been reported to increase due to prophylactic antibiotic use, long hospital stays, open injuries and malnutrition^28,29^. Consistent with this, in our study, 144 strains (40.2%) of MDROs were isolated from the trauma ward, primarily from the infections of incision/wound and urinary tract. It has been indicated previously that the length of hospital stay, duration of antibiotic use, open injury, and serum albumin levels are independent risk factors for multidrug resistant infections in orthopedic trauma patients^22^. This suggests that clinicians should focus on rational use of antibiotics, controlling the length of hospitalization and strengthening the nutritional status of trauma patients, especially those with open injuries in order to prevent multidrug-resistant infections.

In the present study, apart from the trauma ward, the spinal surgery department also provoked our attention. 129 strains of MDROs (36.0%) were isolated from this department, second only to the trauma ward, and it was found that more than half of them were from urinary tract infections. Similarly, Hiroyuki et al.^30^ included 825 spinal surgery patients in their study, and found that the proportion of non-surgical incision infections due to MDROs in these patients was higher than that of surgical incision infections, and urinary tract infections accounted for 87.0% of the patients with non-surgical incision infections. The reasons for these findings may be as follows: (1) high number of invasive spinal procedures requiring catheter placement after surgery, with the indwelling catheter acting as the main cause of urinary tract infection^31,32^; (2) long operation time, older age and large amount of intraoperative fluid input resulting in a higher postoperative urinary retention, which is more likely to induce urinary tract infection^33^. Furthermore, we found that more than 60% of the MDROs isolated from spinal surgery ward were ESBL-positive *E. coli*. The firm binding of pili on the surface of *E. coli* to the urothelial umbrella cells, allows the bacteria to adhere easily to the urinary tract epithelium, preventing them from being washed away by urine^24^. At the same time, factors such as anesthesia, surgery, and long-term bed rest impair the body’s immune system, further contributing to the urinary tract infection. Additionally, it can be inferred that the production of ESBL may be responsible for the increased risk of urinary tract infections in spinal surgery patients and clinicians must pay close attention to this fact. Therefore, in high-risk patients, especially the elderly and paraplegic patients, bladder irrigation should be routinely performed to clean the urethra, dilute the urine and maintain the urinary tract patency to reduce the risk of urinary tract infection. Although the isolation rate of MDROs was low in the other wards, this cannot be overlooked, as infections caused by MDROs frequently lead to serious consequences.

In our study, a total of 68 strains (19.0%) of MRSA were isolated from the department of orthopedics from 2012 to 2017, which is slightly higher than the isolation rates reported by other hospitals in China^14^. MRSA can produce β-lactamase enzyme and hydrolyze antibiotics to achieve drug resistance, therefore it is almost resistant to all β-lactam antibiotics^34^. Consistent with this fact, all MRSA strains isolated in our study were resistant to β-lactam antibiotics, including penicillin, ceftriaxone and ampicillin. Clavulanate and sulbactam are both non-competitive β-lactamase inhibitors, which can bind to β-lactamase secreted by MRSA to form a stable intermediate substrate, thereby reducing the degradation of β-lactamase by antibiotics^35^, thus combining them with β-lactam antibiotics can enhance their antibacterial effect. However, in this study, we found that MRSA showed high resistance rates (> 90%) to amoxicillin/clavulanic acid and ampicillin/sulbactam. These results indicate that the compatibility between β-lactam antibiotics and β-lactam inhibitors is no longer an appropriate factor to consider during treatment of MRSA infections. By contrast, MRSA has a high sensitivity to quinolone antibiotics including levofloxacin, moxifloxacin and ciprofloxacin (drug resistance rate was less than 30%). Although vancomycin-resistant *S. aureus* has been successfully isolated in previous research^36^, no such strains were detected in the present study. Therefore, according to our results, vancomycin can be used for the treatment of MRSA infection in clinical practice. In addition, we found that all MRSA are sensitive to nitrofurantoin and tigecycline, and therefore can also be used as viable options.

ESBL-positive bacteria have been identified as the principal pathogens in nosocomial infections, capable of causing numerous serious clinical complications, as these can destroy the β-lactam ring of antibiotics through the production of ultra-broad-spectrum lactamase (ESBL) and thus make them lose their antibacterial activity, posing a challenge to clinicians in anti-infection treatment^37^. In this study, ESBL-positive bacteria isolated from patients were mainly ESBL-positive *E. coli* (42.2%) and ESBL-positive *K. pneumoniae* (19.0%). As the predominant MDROs, ESBL-positive *E. coli* were found to be sensitive to carbapenem antibiotics like ertapenem (resistance rate = 8.9%), imipenem (resistance rate = 6.6%) and meropenem (resistance rate = 10.6%), indicating that carbapenem antibiotics can still be used as the first-line treatment against the infection of this bacterium^38^. However, due to wide application of carbapenem antibiotics in China, the drug resistance rate of ESBL-positive *E. coli* to carbapenems is gradually increasing. Therefore, finding alternative antibiotics for carbapenems has become a hot topic for clinicals. In recent research, Jesús et al.^39^ demonstrated that carbapenems are not superior to and can be replaced by β-Lactam/β-lactam inhibitor combinations for treating patients with ESBL-positive *E. coli* infection. In our study, we found that ESBL-positive *E. coli* had a lower drug resistance rate to cefoperazone/sulbactam and peracillin/tazobactam compared to carbapenems, with drug resistance rates of 8.4% and 6.5%, respectively. These results suggest that carbapenem antibiotics are still the first-line treatment against ESBL-positive *E. coli* infection, but in some cases, cefoperazone/sulbactam and peracillin/tazobactam might be administered as suitable alternatives. Furthermore, we found that the drug resistance rate of ESBL-positive *K. pneumoniae* was generally higher than that of ESBL-positive *E. coli*, and clinicians need to be wary of this fact. However, it was also found that the resistance patterns of the two bacteria were similar. Therefore, the same treatment strategy can be applied to both type of infections.

Few previous studies have revealed hospital-acquired pneumonia (HAP) as the main nosocomial infection resulting in increased morbidity, mortality, and medical costs^27^. Epidemiologic studies have reported an upward trend in the incidence of HAP attributable to MDROs. Furthermore, MDR nonfermenters (MDR-AB, MDR-PA) have been recognized as the major causative pathogens of HAP in Asia^40^. Therefore, it is essential to be mindful of MDR nonfermenters in relation to HAP in clinical practice, to avoid inappropriate empirical therapy and overuse of antibiotics. In our study, a total of 47 strains (13.1%) of MDR-AB were isolated from the department of orthopedics, and most of these (70%) were isolated from the sputum specimens. Therefore, it can be deduced that MDR-AB was the predominant pathogen causing pulmonary infection in the orthopedics department. Furthermore, we found that MDR-AB showed high resistance to most antibiotics, especially cephalosporins (100%) such as cefotetan, cefazolin, ceftriaxone and ceftazidime. In addition, more than 70% of strains were resistant to quinolones (levofloxacin 78.7%, ciprofloxacin 100%), carbapenems (meropenem 95.5%, imipenem 81.0%) and aminoglycoside antibiotics (amikacin 72.4%, gentamicin 95.7%). Although MDR-AB had the lowest resistance rate to cefoperazone/sulbactam, it still reached 31.7%. Therefore, single drug treatment may not be able to control MDR-AB infection, and combined pharmacotherapy may be a good choice in such cases^41^. Cai et al.^41^ demonstrated that compared with colistin alone, the combination of tigecycline and colistin was an effective therapy for MDR-AB. Besides, this combination therapy can also prevent the emergence of resistance during treatment of MDR-AB infection. Furthermore, thirteen strains (3.6%) of MDR-PA were isolated in our study and drug resistance analysis showed that the resistance of MDR-PA was as severe as that of MDR-AB. Therefore, the combination therapy may also be a good choice for MDR-PA infection^36^.

External factors such as the duration of antibiotic use, ICU admission, and invasive operation have been confirmed by many researchers to be correlated with multidrug-resistant infections^42,43^. However, when it comes to infections caused by MDROs, inherent patient factors are equally important as the external risk factors. In our study, univariate regression analysis was performed on 11 possible risk factors for multidrug-resistant infections, including basic information of the patients (age, gender), personal history (history of smoking, drinking), chronic diseases (hypertension, diabetes), injury type (open injury, central nerve damage) and blood biochemical profile (albumin level, hemoglobin level). It was found that patients with age ≥ 60 years, open injury and central lesion were more prone to infections caused by MDROs. Furthermore, it is noteworthy that multivariate unconditional logistic regression analysis identified these factors as independent risk factors for infections caused by MDROs.

In this study, 31.8% of patients with multidrug-resistant infections were aged ≥ 60 years, which was significantly higher than the proportion of patients without these infections. Previous studies have indicated that elderly patients with impaired immunity and chronic diseases, are at high risk of developing nosocomial infections, especially those caused by MDROs^44^. It is accepted that patients with open wounds are more susceptible to infection because of the lack of intact skin. According to the study of Liang et al.^22^, patients with open injuries often experience serious complications, and demonstrate a higher need for combination antibiotics, long-term antibiotic use, and more frequent admissions to ICU, all of which increase the probability of being infected by MDROs. Therefore, for patients with open injuries, in addition to performing wound debridement as soon as possible, it is crucial to use antibiotics rationally and for a limited duration. The traumatic injuries of central nervous system that are encountered in our orthopedics department mostly involve the spinal cord. As for acute cervical spinal cord injury patients, phrenic nerve can be affected by the spread of edema early on, causing obstruction of patient's breathing, accompanied by cough and weakness. This, coupled with long-term bed rest, can result in the accumulation of a large amount of sputum in the lungs, thus enhancing vulnerability to infections^45^. Critical patients may require tracheotomy and mechanical ventilation due to respiratory inhibition, or even admission to the ICU, all of which are confirmed risk factors for development of multidrug-resistant infections^46^. Furthermore, patients with autonomic nerve injury might lose their normal urination function and need to undergo long-term urethral catheterization, which increases the chances of urinary tract infections with MDROs^47^. Therefore, inpatients with central nervous system injury, clinicians should focus on strengthening the management of respiratory tract and urinary tract while treating the primary disease.

## Conclusion and limitation

In summary, 358 strains of MDROs were isolated from our department of orthopedics, and the detection rate was 25.7%. Among them, ESBL-positive *E. coli* was the most common, followed by MRSA. Trauma and spine are the departments with the highest number of multi-drug resistant bacteria isolated, which need attracted more attention. Besides, drug-resistance patterns of these MDROs demonstrated that antimicrobial resistance remains a serious concern. Notably, doctors must be aware of the infection risk from MDR bacteria. Patients with open injury, central nervous system injury and those aged ≥ 60 years, were more prone to multidrug-resistant infections. Clinicians should pay more attention to such patients in order to actively prevent and control the occurrence of infections caused by MDROs. However, there are still some limitations in this study, as following: (1) This study was a single-centre study and was not compared with the drug resistance of MDROs in other departments, nor was it compared with the infection of MDROs in other hospitals. (2) This study only analyzed the drug resistance of MDROs infected with the top five species, which lacked comprehensiveness. (3) In future studies, we suggest that drug resistance analysis be conducted by different subspecialties to make the results more relevant.

## Data Availability

Data are available from the corresponding author on reasonable request.

## Abbreviations

CLSI: Clinical and Laboratory Standards Institute
ESBL: Extended-spectrum β-lactamase
HAIs: Hospital-acquired infections
HAP: Hospital-acquired pneumonia
MRSA: Methicillin-resistant *Staphylococcus aureus*
MDROs: Multidrug-resistant organisms
MDR-AB: Multidrug-resistant *Acinetobacter baumannii*
MDR-PA: Multidrug-resistant *Pseudomonas aeruginosa*
